# High-Fluidization, Early Strength Cement Grouting Material Enhanced by Nano-SiO_2_: Formula and Mechanisms

**DOI:** 10.3390/ma14206144

**Published:** 2021-10-16

**Authors:** Jiaolong Ren, Zedong Zhao, Yinshan Xu, Siyuan Wang, Haiwei Chen, Jiandong Huang, Boxin Xue, Jian Wang, Jingchun Chen, Chengxu Yang

**Affiliations:** 1 School of Civil and Architectural Engineering, Shandong University of Technology, Zibo 255000, China; worjl@sdut.edu.cn (J.R.); wsy17852030294@163.com (S.W.); chw20000303@163.com (H.C.); xbx03210418@163.com (B.X.); 21507020787@stumail.sdut.edu.cn (J.W.); 21507020774@stumail.sdut.edu.cn (J.C.); 2 School of Transportation and Vehicle Engineering, Shandong University of Technology, Zibo 255000, China; 20402010140@stumail.sdut.edu.cn (Z.Z.); 20502040226@stumail.sdut.edu.cn (C.Y.); 3 Zhejiang Scientific Research Institute of Transport, Hangzhou 310039, China; xys0613@163.com; 4 School of Mines, China University of Mining and Technology, Xuzhou 221116, China

**Keywords:** cement grouting material, formula, hydration mechanisms, high-fluidization, early strength, nano-SiO_2_

## Abstract

Cement grouting material is one of the most important materials in civil construction at present, for seepage prevention, rapid repair, and reinforcement. To achieve the ever-increasing functional requirements of civil infrastructures, cement grouting materials must have the specific performance of high fluidization, early strength, and low shrinkage. In recent years, nanomaterials have been widely used to improve the engineering performance of cement grouting materials. However, the mechanisms of nanomaterials in grouting materials are not clear. Hence, a high-fluidization, early strength cement grouting material, enhanced by nano-SiO_2_, is developed via the orthogonal experimental method in this study. The mechanisms of nano-SiO_2_ on the microstructure and hydration products of the HCGA, in the case of different curing ages and nano-SiO_2_ contents, are analyzed through scanning electron microscopy tests, X-ray diffraction tests, differential scanning calorimetry tests, and Fourier transform infrared spectroscopy tests.

## 1. Introduction

In civil engineering, grouting is one of the most efficient and common methods for seepage prevention, rapid repair, and reinforcement [1,2]. Owing to the advantage in mature technology and satisfactory cost performance, cement-based materials are widely used in grouting [3]. To achieve the ever-increasing functional requirements of civil infrastructures, cement grouting materials must have the following specific characteristics: (a) high fluidization (to ensure that the grouting materials can fill into the defects of the engineering structure easily and fully); (b) early strength (to shorten the engineering period); (c) low shrinkage (to prevent shrinkage cracks at an early age) [4]. In this case, various innovative materials have been used to attempt to prepare modified cement-based grouting materials. Liu et al. [4], Li et al. [5], Li et al. [6], Wu et al. [7], and Zhang et al. [8] adopted aluminate cement, magnesium phosphate cement, sulphoaluminate cement, potassium magnesium phosphate cement, and ultrafine sulphoaluminate cement to improve the early strength and fluidization of cement grouting materials, respectively, which could obtain a significant improvement effect. However, the source of these new types of cements is limited, which might not meet the requirement of engineering applications. Zhou et al. [9], Celik et al. [10], Zhang et al. [11], and Guo et al. [12] adopted water glass, bottom ash, microfine fly ash, and ultrafine cement to modify the fluidization of grouting materials, respectively. However, the three materials found it difficult to improve the strength significantly, especially the early strength. Lu et al. [13], Shi et al. [14], and Zhang et al. [15] adopted sandy pebble soil, glass fiber, and graphene fiber to enhance the early strength of grouting materials, respectively. However, these additives brought less effect on the fluidization. In this case, it is necessary to seek alternative grouting materials to balance high fluidization, early strength, and the material source.

In recent years, nanomaterials have been widely used to improve the performance of cement-based materials because of their large specific surface area, high surface free energy, and good dispersion ability [16]. Feng et al. [17] proved the significance of nano-SiO_2_ on the early age hydration of cement mortars. Qiu et al. [18] revealed the modification effects of nano-CaCO_3_ on the engineering performance of cement grouts. Jiao et al. [19] proved the feasibility of nano-Fe_3_O_4_ used in cement paste. Zhang et al. [20] found that nano-silica could reduce the setting time and increase the early strength of cement composites with a high volume of fly ash. Akono [21] investigated the relationship between nano-TiO_2_ and CSH gel in Portland cement paste. Liu et al. [22], Lang et al. [23], Sargam et al. [24], Ikotun et al. [25], and Ren et al. [26] analyzed the effects of different nanoparticles (e.g., nano-SiO_2_, nano-Al_2_O_3_, nano-CaCO_3_, nano-MgO, nano-TiO_2_, grapheme oxide, and carbon nanotube) on the strength characteristics and engineering performance of different cement-based materials. They considered that the nanomaterials had a similar function for cement-based materials, and nano-SiO_2_ could provide the most significant modification effect. Nano-SiO_2_ can easily bond with the hydration products of cement mortar to generate calcium silicate hydrate gel because of the special network structure of nano-SiO_2_ [27,28]. Sonebi et al. [29] analyzed the effect of the content of nano-SiO_2_ on the rheology, fresh properties, and strength of cement-based grouting materials via the response surface methodology. Unfortunately, the effect of nano-SiO_2_ on the hydration mechanisms is neglected in their study. Zhou et al. [30] investigated the engineering properties and microscopic morphology of cement-based grouting materials modified by nano-SiO_2_. However, the recommended grout in their study is not an early strength material, which is different from the grouting material proposed in our study. Zhang et al. [31] revealed the effects of micro-fine fly ash, colloidal nano-SiO_2_, and superplasticizer on the rheological and mechanical properties of cement-based grouting materials. Although the hydration mechanisms are discussed in their study, the referred mechanisms are not supported by any microscopic tests, and the mechanisms at an early age (e.g., 1 day, 3 days) are neglected. It is reasonable to speculate that nano-SiO_2_ can also modify the engineering performance of cement grouting materials. Although the modification effects of nano-SiO_2_ have been discussed in previous studies, the mechanisms of nano-SiO_2_ on the characteristics of the early strength of cement grouting materials are neglected and not clear. Hence, in this study, a type of high-fluidization, early strength cement grouting material is proposed. The effects of nano-SiO_2_ content and curing age on engineering properties and hydration mechanisms are investigated via macroscopic tests and microscopic tests (i.e., SEM, XRD, DSC, and FTIR), especially for an early age (1 day and 3 days), which is the objective and innovation of this study.

In response to the above issues, a high-fluidization, early strength cement grouting material, enhanced by nano-SiO_2_, is developed via the orthogonal experimental method in this study. Moreover, the mechanisms of nano-SiO_2_ on the microstructure and hydration products, in the case of different curing ages, are analyzed through scanning electron microscopy (SEM) tests, X-ray diffraction (XRD) tests, differential scanning calorimetry (DSC) tests, and Fourier transform infrared spectroscopy (FTIR) tests.

## 2. Materials and Methods

### 2.1. Materials

**Figure 1:**
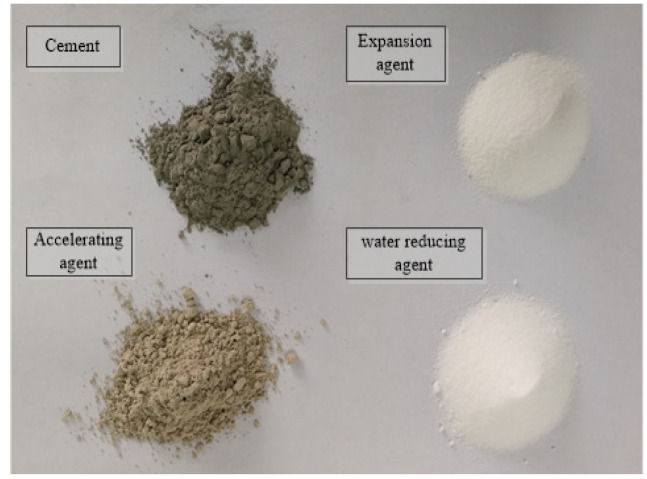
Cement and additives.

**Figure 2:**
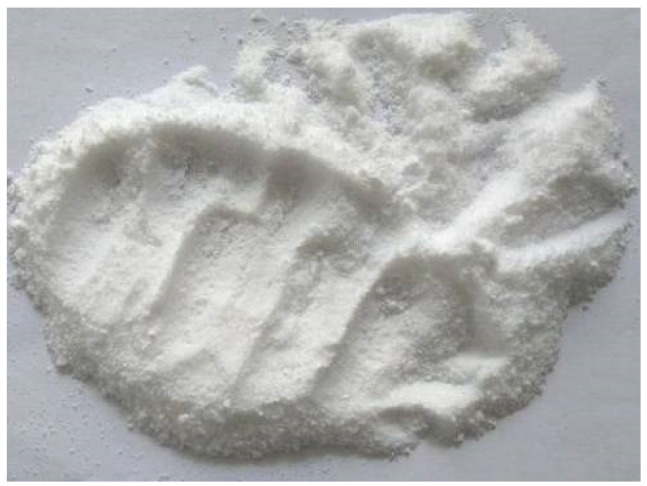
Nano-SiO_2_.

The cement grouting material developed in this paper involves the following five types of raw materials: Shanlv P. O. 42.5R cement, polycarboxylate water-reducing agent, accelerating agent, UEA expansion agent, and nano-SiO_2_, as illustrated in Figure 1 and Figure 2. The technical characteristics of the five types of raw materials are shown in Table 1, Table 2, Table 3, Table 4 and Table 5, respectively.

### 2.2. Methods

In this study, the orthogonal experimental method is used to determine the benchmark formulas of the cement grouting material, owing to the advantage in conveniently analyzing the interrelations among different test factors and scientifically reducing the experimental workload [32,33]. Subsequently, the effects of nano-SiO_2_ content on the engineering performance of the benchmark formulas are analyzed to determine the final high-fluidization, early strength cement grouting material. The fluidity (flowing time), flexural strength (1 day, 3 days, and 7 days), compressive strength (1 day, 3 days, and 7 days), and dry-shrinkage rate (7 days and 28 days) are adopted to evaluate the engineering performance of the cement grouting materials. All the experiments are implemented in accordance with the Chinese specification of “*Test Methods of Cement and Concrete for Highway Engineering*” [34].

Moreover, the SEM (FEI Quanta 250, Anton Paar GmbH, Graz, Austria) test, XRD (AXS, Bruker Corporation, Billerica, USA) test, DSC (SDT 650, TA Instruments, New Castle, USA) test, and FTIR (Nicolet 5700, Thermo Fisher Scientific - CN, Shanghai, China) test are adopted to reveal the mechanisms of the proposed high-fluidization, early strength cement grouting material via microstructure and hydration products. The SEM test is used for the detailed analysis of the micro-morphology of the hydration product. The XRD test is used to investigate the types of hydration products with a scanning speed of 10 °/min and a scanning angle of 10–65° (angle measurement error < 0.01° and angle repeatability < 0.0001°). The DSC test is used to analyze the content of hydration products via the weight change and heat change, ranging from 0 °C to 600 °C, with a heating rate of 15 °C/min (nitrogen atmosphere). The FTIR test is used to investigate functional group characteristics in a spectral range of 400–4000 cm^−1^ in transmission mode using the potassium bromide pressed-disk technique.

The samples used in the SEM tests, XRD tests, DSC tests, and FTIR tests are prepared as follows:

- According to the standard method [34], the beam samples with a size of 4 cm × 4 cm × 16 cm are prepared by curing the target age (1 day, 3 days, or 7 days).
- The sheet samples with a size of 2 cm × 2 cm × 1 cm are prepared by cutting the beam samples, and are put into absolute ethyl alcohol for seven days (the absolute ethyl alcohol must be replaced everyday).
- The treated sheet samples are prepared to cubic blocks with an approximate size of 1 cm × 1 cm × 1 cm after drying at 40 °C for 24 h.
- The SEM samples can be obtained via drying the cubic blocks at 40 °C for 48 h.
- The XRD, DSC, and FTIR samples can be obtained via drying the powder-grinded cubic blocks at 40 °C for 48 h. It should be noted that the powder must be passed through 80 μm, 150 μm, and 80 μm square sieves for the XRD, DSC, and FTIR tests, respectively.

## 3. Optimal Formula of High-Fluidization, Early Strength Cement Grouting Materials

### 3.1. Design of the Orthogonal Experiments

The orthogonal experimental factors and their levels are listed in Table 6. The experimental schemes are presented in Table 7.

### 3.2. Orthogonal Experiment Analysis

The results of the orthogonal experiments are listed in Table 8.

**Figure 3:**
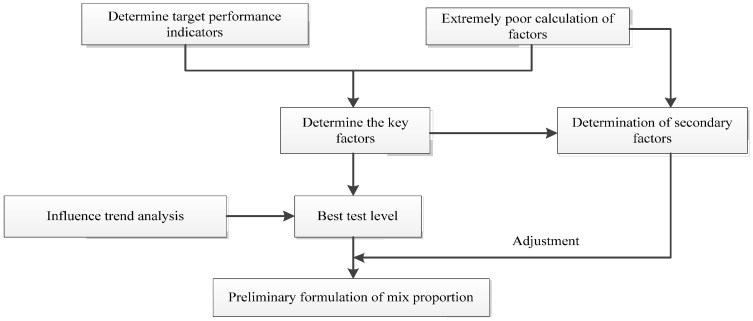
Orthogonal experimental process.

According to Table 8, the ranges for each experimental factor and the corresponding average values for each experimental level are calculated to analyze the orthogonal experimental results, as presented in Table 9. The range is equal to the difference of the average values among different experimental levels for the same experimental factor, as expressed in Equation (1). The influence of the experimental factor increases as the range increases. The process of the orthogonal experimental analysis is shown in Figure 3.

(1) $$RA=TD_{\max}-TD_{\min}$$

where *TD*_max_ and *TD*_min_ are the maximum value and minimum value of the target property index in the case of different experimental levels of a certain experimental factor, respectively.

**Figure 4:**
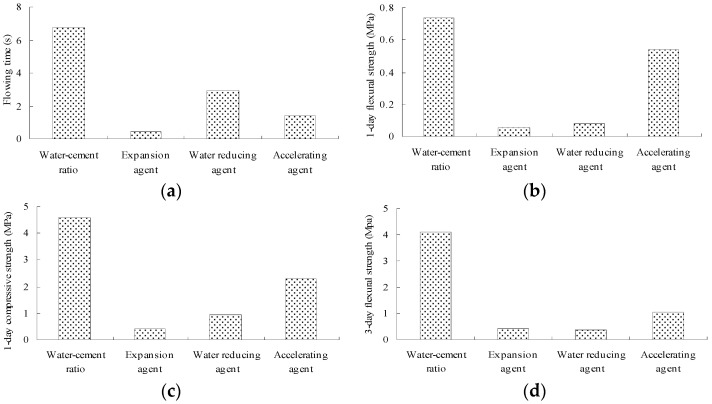

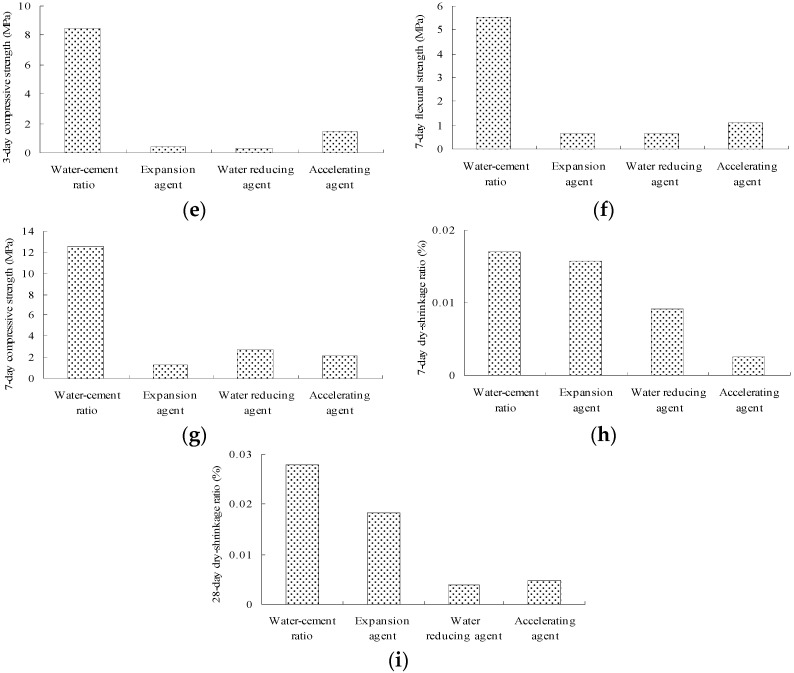
Ranges of different properties. (**a**) Fluidity (flow time); (**b**) 1-day flexural strength; (**c**) 1-day compressive strength; (**d**) 3-day flexural strength; (**e**) 3-day compressive strength; (**f**) 7-day flexural strength; (**g**) 7-day compressive strength; (**h**) 7-day dry-shrinkage ratio; (**i**) 28-day dry-shrinkage ratio.

The ranges of different properties are shown in Figure 4.

According to Figure 3 and Figure 4, the key factor (✔✔) and the secondary factor (✔) for different properties are listed in Table 10.

**Figure 5:**
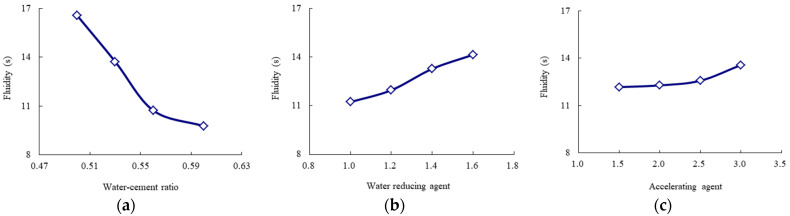
Influence trend of the key and secondary experimental factors for the fluidity (flow time). (**a**) Water–cement ratio; (**b**) water-reducing agent; (**c**) accelerating agent.

**Figure 6:**
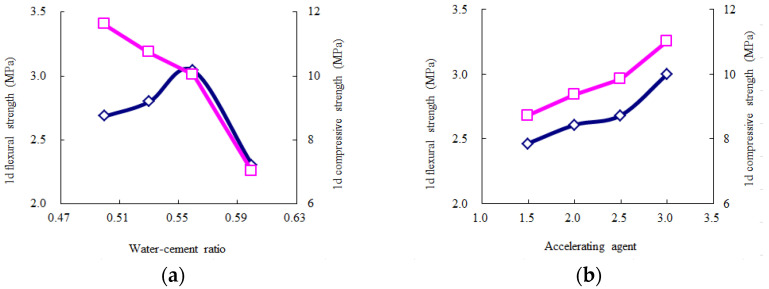
Influence trend of the key and secondary experimental factors for 1-day strength. (**a**) Water–cement ratio; (**b**) accelerating agent.

**Figure 7:**
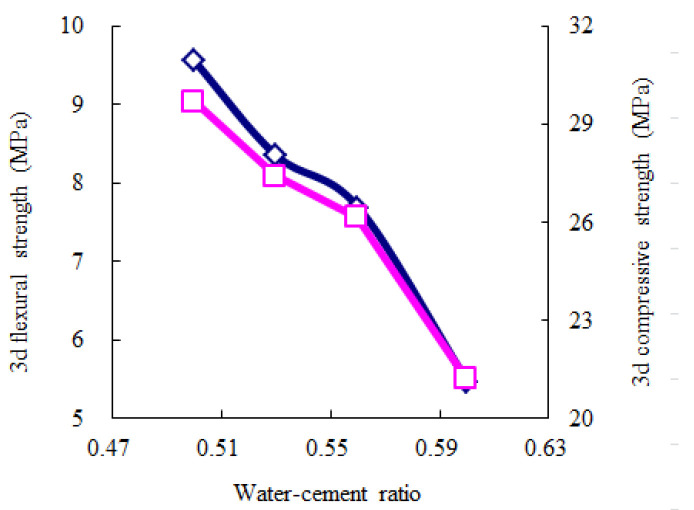
Influence trend of the key experimental factors for 3-day strength.

**Figure 8:**
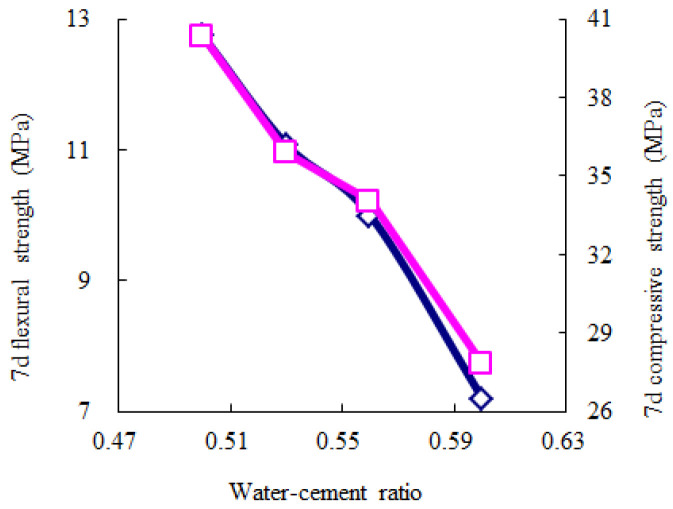
Influence trend of the key experimental factors for 7-day strength.

**Figure 9:**
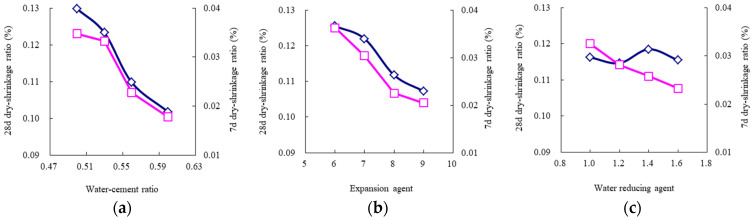
Influence trend of the key and secondary experimental factors for 7-day and 28-day dry-shrinkage ratio. (**a**) Water–cement ratio; (**b**) water-reducing agent; (**c**) accelerating agent.

According to Table 10, the average values for each experimental level can describe the influence trends of the key and secondary experimental factors, as shown in Figure 5, Figure 6, Figure 7, Figure 8 and Figure 9. The blue and red curves correspond to the left and right ordinates, respectively.

As shown in Figure 5, the flow time decreases as the water–cement ratio increases, and the water-reducing agent and accelerating agent decrease. Moreover, when the water–cement ratio is more than 0.56 and the accelerating agent is less than 2.5%, the above trend of the flow time gradually begins to flatten. Hence, considering that the fluidity should range from 9 s to 13 s, according to the Chinese specification “*Technical Specification for Road Semi-Flexible Pavement*” [35], the water–cement ratio is suggested to be more than 0.56, the water-reducing agent is suggested to be less than 1.2%, and the accelerating agent is suggested to be less than 2.5%. As shown in Figure 6, the 1-day compressive strength linearly increases as the accelerating agent increases and the water–cement ratio decreases. Moreover, the 1-day flexural strength increases as the accelerating agent increases; first, it gradually increases and then rapidly decreases as the water–cement ratio increases. When the water–cement ratio is equal to 0.56, the 1-day flexural strength achieves the highest value. Hence, the water–cement ratio is suggested to be 0.53–0.56, and the accelerating agent should be selected as a high level. As shown in Figure 7 and Figure 8, the 3-day and 7-day strengths decrease as the water–cement ratio increases. Considering that a higher strength is better, the water–cement ratio should be selected as a low level. As shown in Figure 9, the 7-day and 28-day dry-shrinkage rates decrease as the water–cement ratio, expansion agent, and water-reducing agent increase. However, when the water–cement ratio and expansion agent are more than 0.56 and 8%, respectively, the descending trend gradually begins to flatten. Hence, the water–cement ratio and expansion agent are suggested to be more than 0.56 and 8%, respectively. The water-reducing agent should be selected as a high level. Note that the optimal proportion of water-reducing agent for the dry-shrinkage rate is contrary to that for the fluidity. However, considering the importance of the water-reducing agent on the fluidity is more significant than the dry-shrinkage rate. The suggested content of water-reducing agent is 1.0%–1.2%. In summary, according to the above analysis of different properties, the effective composition of cement grouting material can be considered to be the following: water–cement ratio = 0.53–0.56, accelerating agent = 2.0%–2.5%, water-reducing agent = 1.0%–1.2%, and expansion agent > 8%.

### 3.3. The High-Fluidization, Early Strength Cement Grouting Enhanced by Nano-SiO_2_

According to the conclusion of Section 3.2, four benchmark formulas are proposed for further verification, as given in Table 11. The results of the engineering performance of the four formulas are presented in Table 12. The performance standard of cement grouting materials shown in Table 12 comes from the Chinese specification “*Technical Specification for Road Semi-Flexible Pavement*” [35].

As shown in Table 12, the fluidity of Y-3 and Y-4 is significantly better than Y-1 and Y-2. Moreover, the flexural strength of Y-4 at an early curing age is higher than Y-3, especially for the 1-day flexural strength. Hence, Y-4 is determined to be the optimal formula.

To further improve the engineering performance, nano-SiO_2_ (see Figure 9) is mixed into the proposed benchmark formula (Y-4). The engineering performance of the cement grouting materials with different contents of nano-SiO_2_ is presented in Table 13. Six specimens are successfully tested for each data. The coefficients of variation (COV) are presented in Table 14. According to the Chinese test specification “*Test Methods of Cement and Concrete for Highway Engineering (JTG E30-2005)*” [34], the COVs of the fluidity, strength, and shrinkage rate must be less than 10%, 10%, and 15%, respectively. It can be found that the COVs all meet the requirements of the Chinese test specification, showing the availability of the test results.

(2) $$COV=\frac{\sigma }{\mu }\times 100\%$$

where *σ* is the standard deviation and *μ* is the average value.

As shown in Table 13, it can be found that the nano-SiO_2_ has a significant effect on the 1-day strength, 3-day strength, and fluidity, especially for the 1-day strength. Every 1% increase in the content of nano-SiO_2_ translates into, on average, a 6.21%, 10.43%, 1.99%, and 3.71% increase in the 1-day flexural strength, 1-day compressive strength, 3-day flexural strength, and 3-day compressive strength, respectively, and translates into a 7.61% fall in the fluidity. This indicates that nano-SiO_2_ can significantly improve the early age strength and slightly weaken the fluidity.

Hence, considering the economy, the formula of the high-fluidization, early strength cement grouting material (HCGA) can be determined as follows: water–cement ratio = 0.56, water-reducing agent = 1.2%, accelerating agent = 2.5%, expansion agent = 8%, and nano-SiO_2_ = 1%.

In addition, it should be noted that, although the chemical nature of nano-SiO_2_ is stable, a possible hazard is breathing in dust because the fine nano-SiO_2_ particles are easy to float in the air. Hence, the handlers must wear masks during construction.

## 4. Hydration Mechanisms of HCGA

The effects of curing age (1-day, 3-day, and 7-day) and nano-SiO_2_ content (0%, 1%, 2%, and 3%) on the microstructure and hydration products of HCGA are analyzed in this section.

### 4.1. Microstructure of HCGA

#### 4.1.1. The Curing Age of 1-Day

**Figure 10:**
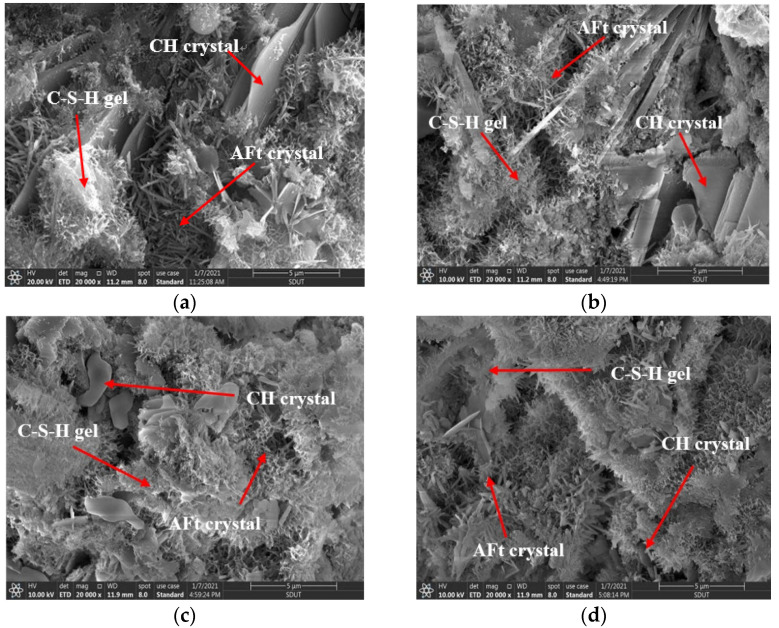
Microstructure of HCGA with different contents of nano-SiO_2_ at 1 d curing age. (**a**) ×20,000, 0%; (**b**) ×20,000, 1%; (**c**) ×20,000, 2%; (**d**) ×20,000, 3%.

The microstructures of the HCGAs with different nano-SiO_2_ contents, at the curing age of 1 day, are shown in Figure 10.

As shown in Figure 10, the following observations can be made.

The CSH (calcium silicate hydrate) gels and AFt crystals (ettringite) can be observed in each HCGA, whether the nano-SiO_2_ is added or not. However, there are some obvious voids in the microstructure of the HCGA without nano-SiO_2_. These voids gradually decrease as the content of nano-SiO_2_ increases. It can be speculated that the nano-SiO_2_ is helpful in improving the hydration of the cement grouting material.

Moreover, the CH(Ca(OH)_2_) crystals provide an effect to guarantee the stable existence of cement hydration products. The CH crystals in the HCGA without nano-SiO_2_ are mainly generated as layered joints at the interface of cement stone, which cannot be wrapped by CSH gels, resulting in restriction of the strength formation. As the content of nano-SiO_2_ increases, the number and size of the layered CH crystals gradually decrease, and the CSH gels accordingly increase, indicating that nano-SiO_2_ is beneficial to accelerate the consumption of CH crystals and the formation of CSH gels. In addition, with the addition of nano-SiO_2_, the CSH gel and AFt crystals are gradually connected to each other, and form an interlaced skeleton structure. The phenomena also explain why the 1-day flexural and compressive strengths of the HCGA increase as the content of nano-SiO_2_ increases.

Hence, it can be speculated that the mechanism of nano-SiO_2_ on the early strength of the HCGA is to accelerate the generation of CH crystals, to reach saturation at a faster rate and urge the CHS gels to generate early, while the mechanism is irrelevant to the AFt crystals. In addition, owing to the accelerated reaction of CH crystals and CHS gels, caused by nano-SiO_2_, the number and size of voids can be effectively controlled.

#### 4.1.2. The Curing Age of 3-Day

**Figure 11:**
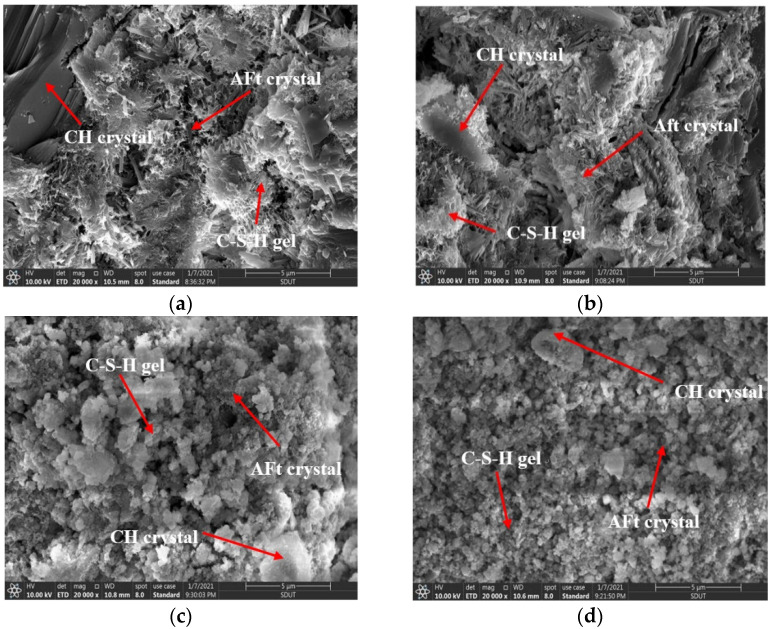
Microstructure of HCGA with different contents of nano-SiO_2_ at 3 d curing age. (**a**) ×20,000, 0%; (**b**) ×20,000, 1%; (**c**) ×20,000, 2%; (**d**) ×20,000, 3%.

The microstructures of the HCGA with different nano-SiO_2_ contents, at the curing age of 3 days, are shown in Figure 11.

As shown in Figure 11, compared to the microstructure at the curing age of 1 day, the number of voids and the amount of layered CH crystals in the HCGA at the curing age of 3 days significantly decreases in the field of the microscope, and the amount of CSH gel accordingly increases. This indicates that the hydration degree of the HCGA is further strengthened. Moreover, as the content of nano-SiO_2_ increases, it can also be found that the CHS gels increase and the layered CH crystals decrease, proving that the effect of nano-SiO_2_ on early hydration still remains. However, the difference in the microstructures in the case of different contents of nano-SiO_2_, at the curing age of 1 day, is less than that at the curing age of 3 days, showing that the effect of nano-SiO_2_ gradually grows less as the curing age increases.

#### 4.1.3. The Curing Age of 7-Day

**Figure 12:**
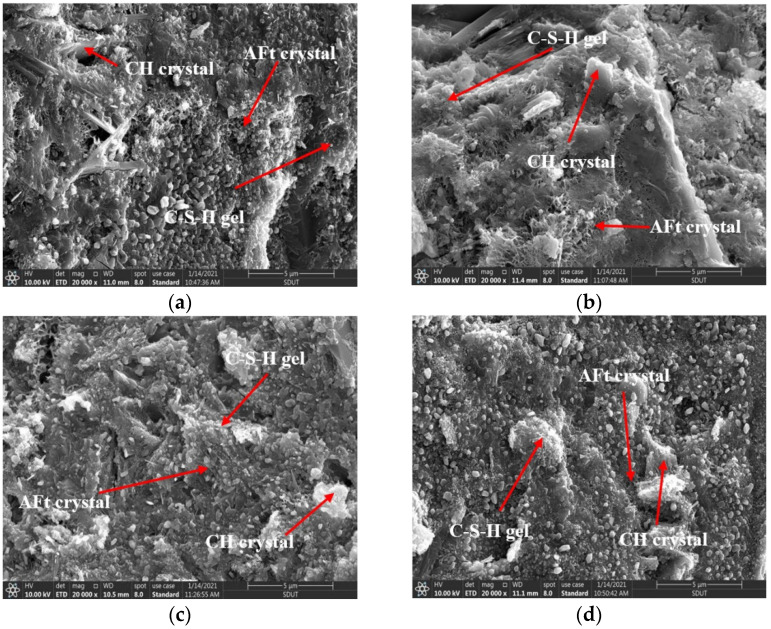
Microstructure of HCGA with different content of nano-SiO_2_ at 7 d age. (**a**) ×20,000, 0%; (**b**) ×20,000, 1%; (**c**) ×20,000, 2%; (**d**) ×20,000, 3%.

The microstructure of cement grouting materials with different nano-SiO_2_ contents at 7 days is shown in Figure 12.

As shown in Figure 12, the hydration products are closely connected to form a relatively dense and stable microstructure. This shows that the hydration of the HCGA has tended to be completed at the curing age of 7 days. In addition, the differences in the microstructure in the case of different contents of nano-SiO_2_ are not significant, indicating that the nano-SiO_2_ has little effect on the hydration of the HCGA at the curing age of 7 days.

In previous studies [15,18], nano-SiO_2_ can also play a significant role in early strength at the curing age of 7 days for common cement-based materials. In contrast, the effect of nano-SiO_2_ weakened at the curing age of 3 days and disappeared at the curing age of 7 days for the HCGA proposed in this study. It can be speculated that the reaction period of nano-SiO_2_ is not fixed, which is related to the hydration rate. The effect of nano-SiO_2_ on the strength will occur ahead, as the hydration rate quickens.

### 4.2. X-ray Diffraction Analysis

**Figure 13:**
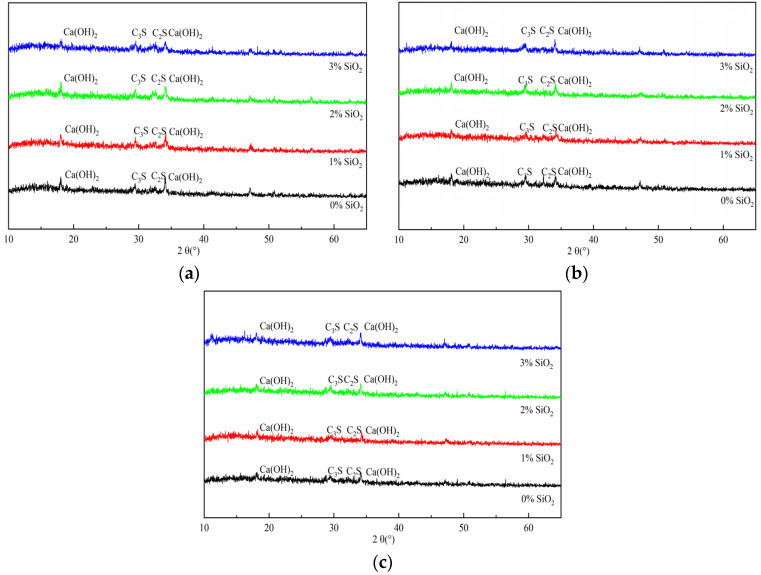
XRD images. (**a**) 1 day; (**b**) 3 days; (**c**) 7 days.

Figure 13 shows the XRD results of the HCGA in the case of different contents of nano-SiO_2_ at the curing age of 1 day, 3 days, and 7 days. In Figure 13, C_2_S and C_3_S represent dicalcium silicate and tricalcium silicate, respectively.

As shown in Figure 13, the constituents of the HCGA in the case of different contents of nano-SiO_2_ are similar in the XRD images. At the curing age of 1 day, the intensity of the diffraction peak of C_3_S decreases as the content of nano-SiO_2_ increases, showing that nano-SiO_2_ accelerates the consumption of C_3_S to generate CH crystals and CSH gels, to realize the early strength. When the diffraction angle is 35°, the changes in the CH crystals are similar to when the diffraction angle is 28° [36,37], owing to the formation of the CSH gels, caused by the reaction of nano-SiO_2_ and CH crystals. This is the reason that the diffraction peak of CH crystals decreases as the content of nano-SiO_2_ increases. In addition, the differences in the derivative peak of C_2_S in the case of different contents of nano-SiO_2_ are limited, showing that nano-SiO_2_ has little effect on the long-term strength of the HCGA. The above phenomena show that nano-SiO_2_ mainly takes part in the hydration reaction of C_3_S to improve the early strength in the HCGA, while it is irrelevant to the C_2_S.

Moreover, the diffraction peaks at the curing age of 3 days and 7 days are similar to those at the curing age of 1 day, indicating that there is no new hydration reaction during the curing age of 3 days and 7 days. The intensities of the diffraction peaks of C_2_S and C_3_S decrease as the curing age increases. This implies that the hydration of the HCGA is still ongoing at the curing age of 3 days and 7 days. In addition, the difference in the diffraction peaks in the case of different contents of nano-SiO_2_ at the curing age of 7 days shows that nano-SiO_2_ has little effect on hydration at the curing age of 7 days.

### 4.3. Differential Scanning Calorimetry

**Figure 14:**
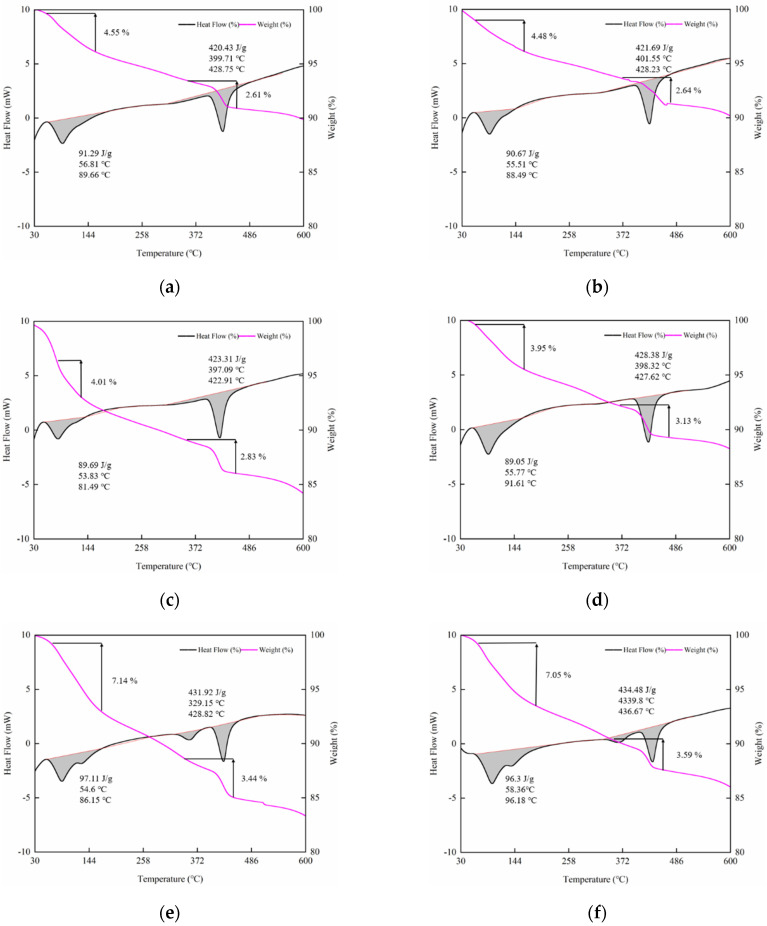

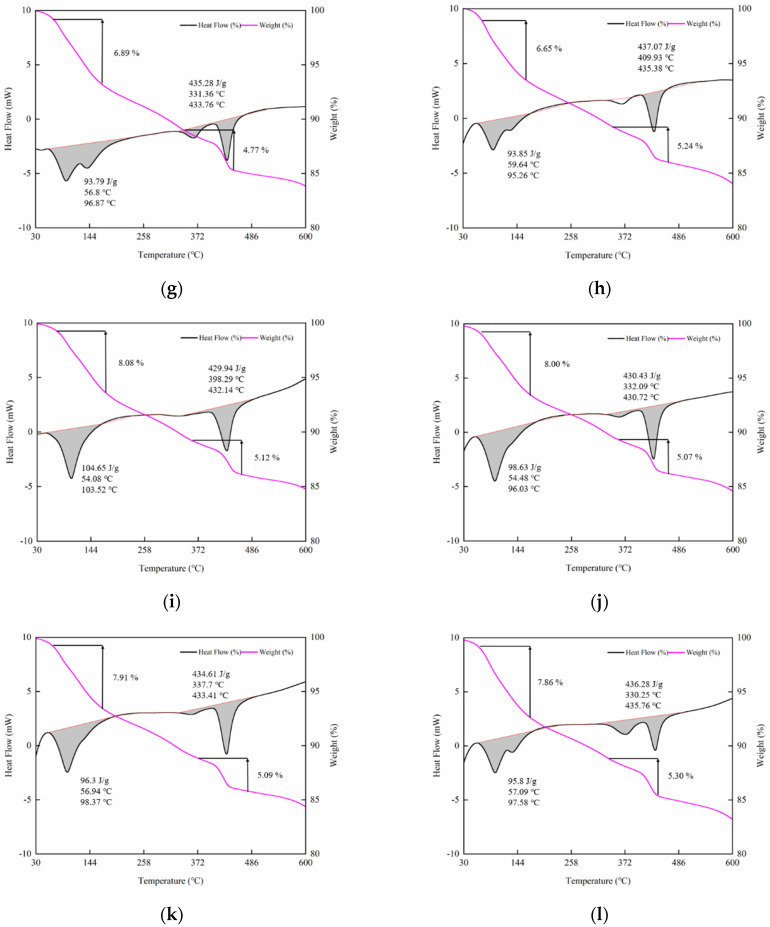
DSC-TG curve. (**a**) 1 day, 0% SiO_2_; (**b**) 1 day, 1% SiO_2_; (**c**) 1 day, 2% SiO_2_; (**d**) 1 day, 3% SiO_2_; (**e**) 3 days, 0% SiO_2_; (**f**) 3 days, 1% SiO_2_; (**g**) 3 days, 2% SiO_2_; (**h**) 3 days, 3% SiO_2_; (**i**) 7 days, 0% SiO_2_; (**j**) 7 days, 1% SiO_2_; (**k**) 7 days, 2% SiO_2_; (**l**) 7 days, 3% SiO_2_.

**Figure 15:**
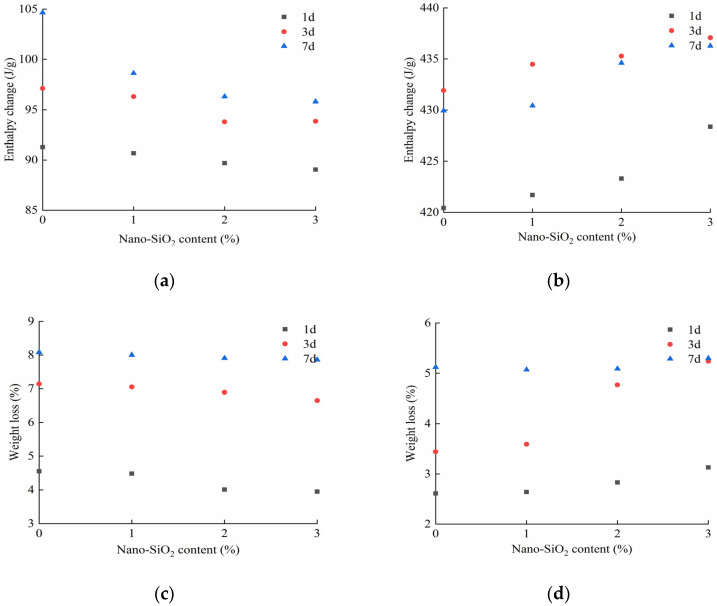
(**a**) Enthalpy change in the I-stage stage; (**b**) enthalpy change in the II-stage stage; (**c**) weight loss in the I-stage stage; (**d**) weight loss in the II-stage stage.

The mass loss curve (TG curve, red) and heat flow curve (DSC curve, black) of the HCGA are shown in Figure 14. In the curves, there are two obvious segments for the weight loss and enthalpy change. The first thermal decomposition peak and the corresponding weight loss that appeared at lower than 150 °C mainly represent the evaporation of free water [38], abbreviated as I-stage. The second thermal decomposition peak and the corresponding weight loss that appeared at 350–600 °C represent the decomposition of CH crystals [39], abbreviated as II-stage. Moreover, the enthalpy change and weight loss in the DSC curves are extracted to further analyze the effects of nano-SiO_2_ content and curing age, as shown in Figure 15.

As shown in Figure 14 and Figure 15, at the curing age of 1 day and 3 days, the weight loss and enthalpy change in the I-stage decrease by 3.34% and 0.97%, on average, for every 1% increase in the content of nano-SiO_2_, respectively, while, in the II-stage, they accordingly increase by 12.04% and 0.51%. The less free water there is, the more bound water there is, and the more complete the hydration reaction is. This implies that nano-SiO_2_ promotes the hydration reaction of the HCGA at an early curing age. Moreover, the increase in weight loss in the II-stage indicates the accelerated generation of CH crystals. This shows that nano-SiO_2_ is conducive, to accelerate the generation of CH crystals to reach saturation at a faster rate, verifying the conjecture in Section 4.1.1. In addition, the change in weight loss and enthalpy change at the curing age of 3 days is, on average, 35.83% and 5.33% less than that at the curing age of 1 day, respectively, implying that the effect of nano-SiO_2_ on the hydration reaction at the curing age of 3 day is lower than that at the curing age of 1 day. When the curing age is 7 days, the difference in the weight loss and enthalpy change in the case of different contents of nano-SiO_2_ is not significant, showing that nano-SiO_2_ has little influence at the curing age of 7 days.

In addition, peak-splitting, for both the observed peaks, can be found in some DSC curves. The DSC curve obtained by the chemical reaction should be a single smooth peak under ideal test conditions. However, the peak shape may be deformed, resulting from overlapping reactions in the process of sample preparation and testing, owing to the unevenness of raw materials, the uncertainty of cement hydration, and the thermal decomposition reaction in an inert atmosphere. Moreover, considering the aging of the apparatus used in this study, the above phenomenon is more significant.

### 4.4. Fourier Transform Infrared Spectroscopy

**Figure 16:**
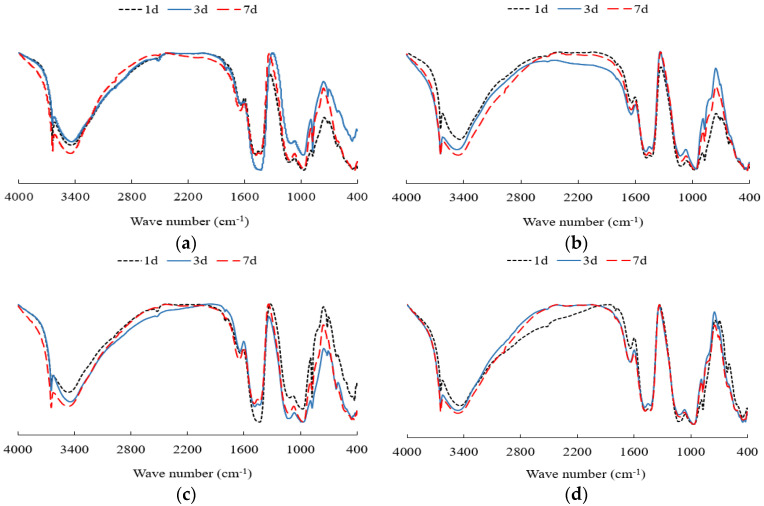
FTIR spectrums. (**a**) SiO_2_ = 0%; (**b**) SiO_2_ = 1%; (**c**) SiO_2_ = 2%; (**d**) SiO_2_ = 3%.

The results of the FTIR tests are shown in Figure 16.

The vibration peak mainly corresponds to the water molecules and Si-O-T (T = Si and Al) in CSH gels. At 4000–400 cm^−1^, the FTIR vibration bands of the HCGA with different contents of nano-SiO_2_ are almost the same. The peak values of tensile vibration and flexural vibration of bound water also do not change significantly. This indicates that the types of hydration products are the same in the case of different curing ages and nano-SiO_2_ contents. The absorption peak at 3643–3645 cm^−1^ is caused by the –OH stretching vibration of Ca(OH)_2_ [40,41]. It can be found that the wave number slightly increases as the content of nano-SiO_2_ increases, showing that the bond energy of –OH in Ca(OH)_2_ is improved; that is to say that the amount of CH crystals increases as the content of nano-SiO_2_ increases. This is consistent with the aforementioned analysis on the hydration process. In addition, the absorption peak at 1639–1646 cm^−1^ is due to the bending vibration caused by –OH in water molecules. The absorption peak at 1480–1485 cm^−1^ is due to the CO_3_^2−^ antisymmetric stretching vibration. This implies that the calcium hydroxide in the cement grout reacts with the carbon dioxide in the air to form calcium carbonate during the preparation of the samples. The range of 400–1400 cm^−1^ is generally identified as a fingerprint area.

## 5. Conclusions

A high-fluidization, early strength cement grouting material, enhanced by nano-SiO_2_ (HCGA), is developed via the orthogonal experimental method in this study. Moreover, the mechanisms of nano-SiO_2_ on the microstructure and hydration products, in the case of different curing ages and nano-SiO_2_ contents, are analyzed through SEM tests, XRD tests, DSC tests, and FTIR tests.

- The formula of the HCGA is water–cement ratio = 0.56, water-reducing agent = 1.2%, accelerating agent = 2.5%, expansion agent = 8%, and nano-SiO_2_ = 1%. The flexural and compressive strength of the HCGA at the curing age of 1 day is higher than 3.5 MPa and 12 MPa, respectively, while the fluidity and shrinkage rate is less than 11 s and 0.15%, respectively;
- Nano-SiO_2_ can significantly improve the flexural and compressive strength of the HCGA at an early curing age, while it will slightly weaken the fluidity. The enhancement of nano-SiO_2_ on the strength becomes weak when the content of nano-SiO_2_ exceeds 1%. Hence, considering economic costs, it is recommended that the recommended content of nano-SiO_2_ is 2%. In addition, the effects of nano-SiO_2_ decrease as the curing age increases, which has little significance at the curing age of 7 days. The mechanism of nano-SiO_2_ on the early strength of the HCGA is to accelerate the generation of CH crystals, to reach saturation at a faster rate and urge the CHS gels to generate early, while it is irrelevant to the AFt crystals;
- The types of hydration products of the HCGA are almost the same in the case of different curing ages and nano-SiO_2_ contents. Nano-SiO_2_ mainly takes part in the hydration reaction of tricalcium silicate, to improve the early strength in the HCGA, while it is irrelevant to the dicalcium silicate. The reaction period of nano-SiO_2_ is not fixed, which is related to the hydration rate. Compared to common cement-based materials, the effect of nano-SiO_2_ on the strength will occur ahead, as the hydration rate quickens in the HCGA (early strength materials).

In addition, thermogravimetric analysis can be used for the quantitive analysis of hydration products. Owing to the limitation of the obtained data in this study, more in-depth quantitative analysis, based on DSC tests, will be addressed in future studies.

## Figures and Tables

**Table 1:** Technical characteristics of cement.

Stability	Setting Time (Min)		Flexural Strength (MPa)		Compressive Strength (MPa)	
	Initial Setting	Permanent Setting	3d	28d	3d	28d
Qualification	170	210	5.7	8.9	30	53.6

**Table 2:** Technical characteristics of water-reducing agent.

Water Reduction (%)	Density (g/cm^3^)	Chloride Ion Content (%)	Alkali Content (%)	Bleeding Rate (%)	Compressive Strength Ratio (%)	
					7d	28d
21.2	1.031	0.21	3.5	30	150	135

**Table 3:** Technical characteristics of accelerating agent.

Setting Time (Min)		Fineness (%)	Water Content (%)	28d Compressive Strength Ratio (%)	1d Compressive Strength (MPa)
Initial Setting	Permanent Setting				
2–3	8–10	11.6	1.7	75	9

**Table 4:** Technical characteristics of expansion agent.

Chemical Composition				Fineness		
Magnesium Oxide (%)	Water Content (%)	Total Alkali Content (%)	Chloride Ion (%)	Specific Surface Area (m^2^·kg^−1^)	0.08 mm Material Retained (%)	1.25 mm Material Retained (%)
2.661	0.80	0.15	0.01	333	7.0	0.31

**Table 5:** Technical characteristics of nano-SiO_2_.

Particle Size (nm)	Specific Surface Area (m^2^/g)	Bulk Density (g/cm^3^)	Purity (%)	Appearance
15	600	0.21	99.8	White grainy

**Table 6:** Experimental factors and experimental levels.

Level	Water–Cement Ratio	Expansion Agent (%)	Water-Reducing Agent (%)	Accelerating Agent (%)
I	0.50	6	1.0	1.5
II	0.53	7	1.2	2.0
III	0.56	8	1.4	2.5
IV	0.60	9	1.6	3.0

**Table 7:** Experimental schemes.

No.	Water–Cement Ratio	Expansion Agent (%)	Water-Reducing Agent (%)	Accelerating Agent (%)
1	0.50	6	1.0	1.5
2	0.50	7	1.2	2.0
3	0.50	8	1.4	2.5
4	0.50	9	1.6	3.0
5	0.53	8	1.0	2.0
6	0.53	9	1.2	1.5
7	0.53	6	1.4	3.0
8	0.53	7	1.6	2.5
9	0.56	9	1.0	2.5
10	0.56	8	1.2	3.0
11	0.56	7	1.4	1.5
12	0.56	6	1.6	2.0
13	0.60	7	1.0	3.0
14	0.60	6	1.2	2.5
15	0.60	9	1.4	2.0
16	0.60	8	1.6	1.5

**Table 8:** Results of the orthogonal experiments.

No.	Flowing Time (s)	Flexural Strength (MPa)			Compressive Strength (MPa)			Shrinkage Rate (%)	
		1-Day	3-Day	7-Day	1-Day	3-Day	7-Day	7-Day	28-Day
1	14.07	2.46	9.12	12.99	10.31	28.97	40.01	0.047	0.138
2	14.68	2.61	9.37	11.64	10.88	29.25	38.62	0.038	0.133
3	17.46	2.79	9.73	12.64	12.65	30.14	38.97	0.030	0.127
4	19.98	2.88	10.01	13.73	12.61	30.39	43.87	0.024	0.121
5	12.82	2.72	8.55	10.70	11.13	27.58	34.88	0.035	0.117
6	13.06	2.61	7.79	10.68	9.62	26.23	35.67	0.027	0.111
7	14.67	3.05	8.89	11.62	12.05	28.59	36.02	0.041	0.139
8	14.34	2.81	8.17	11.31	10.11	27.26	37.00	0.030	0.126
9	9.22	3.11	8.07	10.86	11.01	26.19	34.91	0.021	0.101
10	10.84	3.40	8.21	10.01	10.46	26.67	34.39	0.017	0.107
11	11.09	2.76	7.21	9.12	9.29	25.68	31.4	0.022	0.112
12	11.76	2.91	7.28	9.96	9.38	26.01	35.39	0.031	0.119
13	8.78	2.67	6.17	8.24	8.97	21.76	28.76	0.027	0.109
14	9.96	2.31	5.48	7.06	7.29	21.75	28.70	0.026	0.106
15	9.85	2.21	5.25	6.94	6.11	20.69	26.54	0.010	0.096
16	10.46	2.03	5.01	6.54	5.68	20.67	27.4	0.008	0.096

**Table 9:** Extreme deviation calculation of orthogonal experiment.

Index			Water–Cement Ratio	Water-Reducing Agent	Accelerating Agent	Expansion Agent
Fluidity (Flowing time) (s)	Average value	level I	16.55	11.22	12.17	12.62
		level II	13.72	11.95	12.28	13.09
		level III	10.73	13.27	12.57	12.90
		level IV	9.76	14.14	13.58	13.03
	Range		6.79	2.91	1.40	0.47
1-day flexural strength (MPa)	Average value	level I	2.69	2.74	2.47	2.68
		level II	2.80	2.73	2.61	2.71
		level III	3.04	2.70	2.68	2.74
		level IV	2.30	2.66	3.00	2.70
	Range		0.74	0.09	0.54	0.06
1-day compressive strength (MPa)	Average value	level I	11.61	10.36	8.73	9.76
		level II	10.73	9.72	9.37	10.17
		level III	10.04	10.02	9.86	9.98
		level IV	7.01	9.44	11.02	9.83
	Range		4.60	0.91	2.30	0.41
3-day flexural strength (MPa)	Average value	level I	9.56	7.98	7.28	7.69
		level II	8.35	7.77	7.61	8.10
		level III	7.69	7.77	7.72	7.88
		level IV	5.48	7.62	8.32	7.78
	Range		4.08	0.36	1.04	0.40
3-day compressive strength (MPa)	Average value	level I	29.69	26.13	25.39	26.33
		level II	27.41	26.01	25.88	25.99
		level III	26.13	26.28	26.10	26.27
		level IV	21.22	26.08	26.85	25.88
	Range		8.47	0.27	1.47	0.46
7-day flexural strength (MPa)	Average value	level I	12.75	10.70	9.83	10.41
		level II	11.08	10.02	9.81	10.61
		level III	9.99	10.08	10.25	9.97
		level IV	7.20	10.39	10.90	10.55
	Range		5.56	0.68	1.09	0.64
7-day compressive strength (MPa)	Average value	level I	40.37	34.64	33.62	35.03
		level II	35.89	34.40	33.86	35.23
		level III	34.02	33.23	34.51	33.91
		level IV	27.85	35.92	35.76	35.25
	Range		12.51	2.68	2.14	1.34
7-day shrinkage rate (%)	Average value	level I	0.035	0.033	0.026	0.036
		level II	0.033	0.028	0.029	0.030
		level III	0.023	0.026	0.027	0.023
		level IV	0.018	0.023	0.027	0.021
	Range		0.017	0.009	0.003	0.016
28-day shrinkage rate (%)	Average value	level I	0.130	0.116	0.114	0.126
		level II	0.123	0.115	0.116	0.122
		level III	0.110	0.119	0.115	0.112
		level IV	0.102	0.116	0.119	0.107
	Range		0.028	0.004	0.005	0.018

**Table 10:** The key factor and the secondary factor for different properties.

Property Index	Water–Cement Ratio	Expansion Agent	Water-Reducing Agent	Accelerating Agent
Fluidity (flow time)	✔✔		✔	✔
1-day flexural strength	✔✔			✔
1-day compressive strength	✔✔			✔
3-day flexural strength	✔✔			
3-day compressive strength	✔✔			
7-day flexural strength	✔✔			
7-day compressive strength	✔✔			
7-day dry-shrinkage ratio	✔✔	✔✔	✔	
28-day dry-shrinkage ratio	✔✔	✔		

**Table 11:** Test scheme.

No.	Water–Cement Ratio	Water-Reducing Agent	Accelerating Agent	Expansion Agent
Y-1	0.53	1.0%	2.0%	9%
Y-2	0.53	1.0%	2.5%	9%
Y-3	0.56	1.2%	2.0%	8%
Y-4	0.56	1.2%	2.5%	8%

**Table 12:** Test results.

No.	Fluidity (s)	Flexural Strength (MPa)			Compressive Strength (MPa)			Shrinkage Rate (%)	
		1-Day	3-Day	7-Day	1-Day	3-Day	7-Day	7-Day	28-Day
Y-1	12.86	2.69	8.35	11.10	10.82	27.11	35.89	0.018	0.121
Y-2	13.11	2.89	8.46	11.21	11.39	27.29	35.91	0.027	0.118
Y-3	10.01	3.13	8.06	10.35	10.29	25.86	34.17	0.019	0.101
Y-4	10.38	3.43	8.18	10.66	10.91	26.02	34.38	0.021	0.108
Requirement	9–13	--	--	≥ 2	--	--	10–30	--	<0.5

**Table 13:** Performance of cement mortar with different nano-SiO_2_ contents.

Nona-SiO_2_ Content	Fluidity (s)	Flexural Strength (MPa)			Compressive Strength (MPa)			Shrinkage Rate (%)	
		1-Day	3-Day	7-Day	1-Day	3-Day	7-Day	7-Day	28-Day
0%	10.38	3.43	8.18	10.66	10.91	26.02	34.38	0.019	0.108
1%	10.61	3.77	8.46	10.68	12.18	27.16	35.01	0.017	0.111
2%	11.29	3.98	8.59	10.76	13.01	28.38	34.69	0.021	0.108
3%	12.89	4.11	8.68	10.61	14.68	29.02	34.61	0.019	0.113
Standard	9–13	--	--	≥ 2	--	--	10–30	--	<0.5

**Table 14:** COV of performance of cement mortar with different nano-SiO_2_ contents.

Nona-SiO_2_ Content	COV (%)								
	Fluidity	Flexural Strength			Compressive Strength			Shrinkage Rate	
		1-Day	3-Day	7-Day	1-Day	3-Day	7-Day	7-Day	28-Day
0%	5.29	8.63	3.31	4.38	7.48	4.88	3.29	7.38	2.31
1%	7.31	3.52	4.87	3.45	4.62	6.20	5.13	10.89	4.98
2%	4.26	5.96	6.66	2.96	9.04	6.34	5.67	5.02	3.70
3%	8.60	7.29	5.88	3.77	5.11	3.07	4.74	9.97	2.11
Requirement	<10	<10						<15	

## Data Availability

The data presented in this study are available in article.
